# Acetol biosynthesis enables NADPH balance during nitrogen limitation in engineered *Escherichia coli*

**DOI:** 10.1186/s12934-025-02687-z

**Published:** 2025-03-16

**Authors:** Suresh Sudarsan, Philipp Demling, Emre Ozdemir, Aziz Ben Ammar, Philip Mennicken, Joerg M. Buescher, Guido Meurer, Birgitta E. Ebert, Lars M. Blank

**Affiliations:** 1 Institute of Applied Microbiology (iAMB), Aachen Biology and Biotechnology (ABBt), RWTH Aachen University, 52074 Aachen, Germany; 2 The Novo Nordisk Foundation Center for Biosustainability, Technical University of Denmark, Kemitorvet 220, 2800 Kgs. Lyngby, Denmark; 3 BRAIN Biotech AG, 64673 Zwingenberg, Germany; 4 Present Address: Max Planck Institute of Immunobiology and Epigenetics, 79108 Freiburg, Germany; 5 Australian Institute for Bioengineering and Nanotechnology, The University of Queensland, St Lucia, QLD 4072 Australia

**Keywords:** Metabolic engineering, ^13^C metabolic flux analysis, Glycerol, Acetol, *Escherichia coli*, Cofactor generation

## Abstract

**Background:**

Nutrient limitation strategies are commonly applied in bioprocess development to engineered microorganisms to further maximize the production of the target molecule towards theoretical limits. Biomass formation is often limited under the limitation of key nutrients, and understanding how fluxes in central carbon metabolism are re-routed during the transition from nutrient excess to nutrient-limited condition is vital to target and tailor metabolic engineering strategies. Here, we report the physiology and intracellular flux distribution of an engineered acetol-producing *Escherichia coli* on glycerol under nitrogen-limited, non-growing production conditions.

**Results:**

Acetol production in the engineered *E. coli* strain is triggered upon nitrogen depletion. During nitrogen limitation, glycerol uptake decreased, and biomass formation rates ceased. We applied ^13^C-flux analysis with 2-^13^C glycerol during exponential growth and nitrogen starvation to elucidate flux re-routing in the central carbon metabolism. The results indicate a metabolically active non-growing state with significant flux re-routing towards acetol biosynthesis and reduced flux through the central carbon metabolism. The acetol biosynthesis pathway is favorable for maintaining the NADPH/NADP^+^ balance.

**Conclusion:**

The results reported in this study illustrate how the production of a value-added chemical from a waste stream can be connected to the metabolism of the whole-cell biocatalyst, making product formation mandatory for the cell to maintain its NADPH/NADP^+^ balance. This has implications for process design and further metabolic engineering of the whole-cell biocatalyst.

**Supplementary Information:**

The online version contains supplementary material available at 10.1186/s12934-025-02687-z.

## Background

The envisaged circular bioeconomy can contribute to a reduced carbon footprint, especially when major carbon dioxide-emitting crude oil products, including liquid fuels, can be (partially) replaced with bio-based, low-emitting alternatives. For diesel engines, blending biodiesel from renewable resources such as used cooking oil is attractive; however, its production leads to significant amounts of glycerol as a side-product (10% v_glycerol_/v_biodiesel_) [1]. Due to its increased availability, intrinsic properties like a higher degree of reduction than glucose [2], and its liquid nature at room temperature, glycerol is discussed as a carbon source for industrial biotechnology [3–5].

*Escherichia coli* is a potential candidate for glycerol-based bioprocesses because it can naturally grow on glycerol as its sole energy and carbon source. The integral membrane protein aquaglyceroporin (GlpF) is a glycerol transport facilitator, increasing the diffusion rate over the cytoplasmic membrane [6]. In the cytoplasm, glycerol is activated by the glycerol kinase (GlpK), forming glycerol-3-phosphate (G3P) [7, 8]. This enzyme determines the flux of glycerol metabolism in *E. coli* as it is non-competitively feedback inhibited by fructose-1,6-biphosphate. Under aerobic conditions, a homodimeric glycerol-3-phosphate dehydrogenase converts G3P to dihydroxyacetone phosphate (DHAP), accepting nitrate or oxygen as an electron acceptor [9]. As DHAP is an intermediate of the Embden–Meyerhof–Parnas pathway, further conversion of DHAP proceeds via the central carbon metabolism of *E. coli* [10, 11]. To valorize glycerol by using it as the sole carbon source, its uptake rate has to be high, which is often achieved by adaptive laboratory evolution (ALE) in *E*. *coli* [12, 13] and *S*. *cerevisiae* [14, 15].

A prominent example of an industrially relevant product potentially produced from glycerol is acetol or hydroxy acetone. Acetol is a simple hydroxyketone used as an intermediate for polyol and acrolein synthesis. Further, acetol is applied in the textile industry as a substitute for sodium dithionite [16] and in the cosmetic industry as a skin tanning agent [17–19]. Chemical synthesis of acetol is, for example, achieved by the dehydrogenation of propylene glycol [20]. However, non-perfect selectivity and, hence, cost are drivers to produce acetol through a biological and environmentally friendly route [18].

**Fig. 1:**
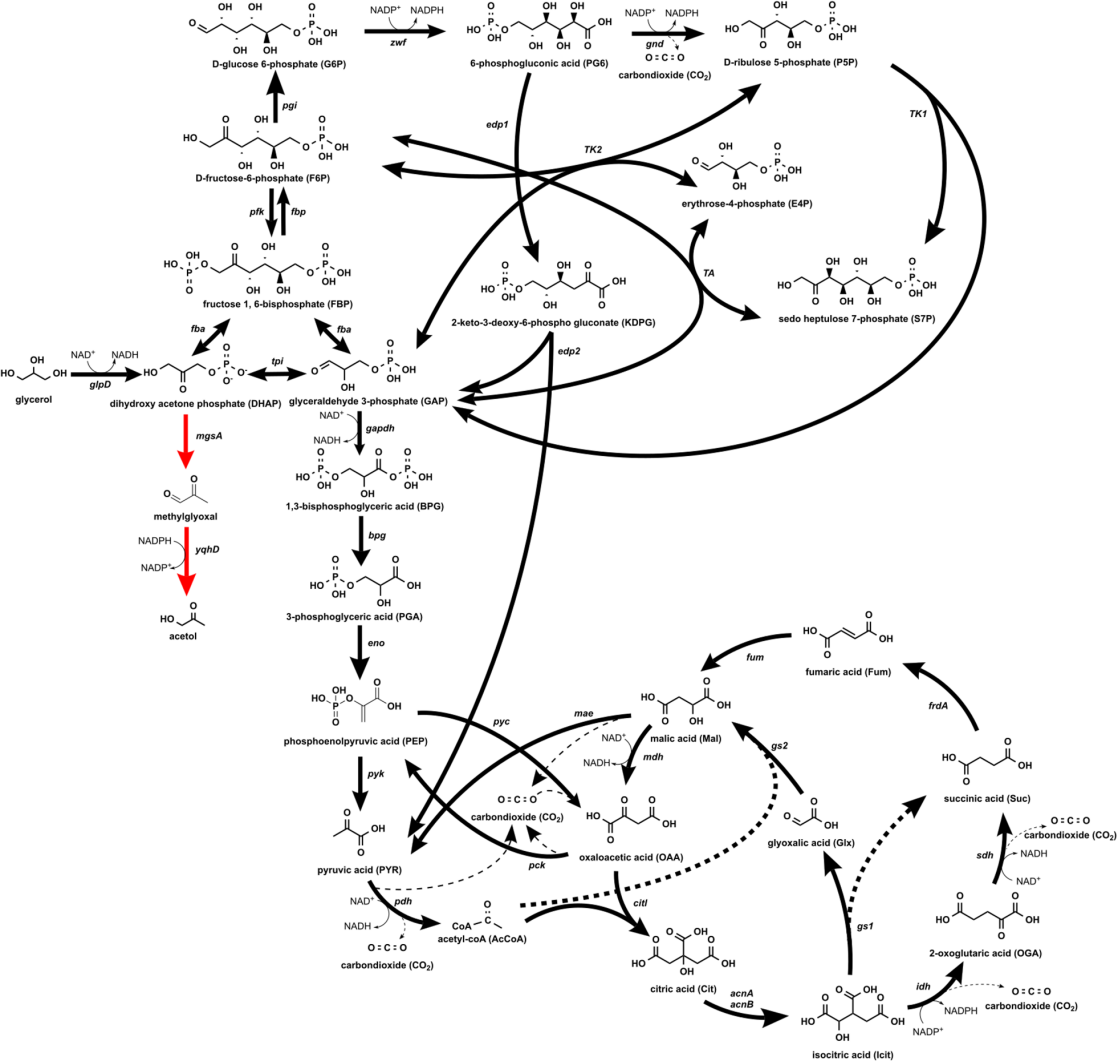
Central carbon metabolism involved in converting glycerol to acetol in *E. coli* B4. The heterologous pathway for acetol production is highlighted in red

Through the methylglyoxal synthase (MGS), which is encoded by the *mgsA* gene, DHAP can be converted to methylglyoxal [21–23]. The NADPH-dependent aldehyde oxidoreductase (AOR, *yqhD*) further catalyzes the reaction from methylglyoxal to acetol (Fig. 1). Using glycerol as a sole carbon source poses a challenge if an NADPH-dependent production. Therefore, different approaches applying metabolic engineering of *E. coli* to enhance co-factor availability and boost the production of acetol and its derivatives have been performed [24–28]. Thereby, substantial titers of 2.8 g L^−1^ acetol solely from 10 g L^−1^ glycerol [27] or 5.6 g L^−1^ 1,2-propanediol from 10 g L^−1^ glycerol in medium supplemented 10 g L^−1^ tryptone and 5 g L^−1^ yeast extract [25] have been reached.

Here, we report the uncoupling of growth from acetol production via nitrogen limitation. The metabolic changes during the shift from nitrogen excess to nitrogen-limiting conditions were studied using carbon isotope labeling experiments. 2-^13^C-labeled glycerol was used as the sole carbon source to monitor the changes in labeling patterns of key intracellular metabolites and the incorporation of labeled carbon into the proteinogenic amino acids. The results indicate a substantial flux redistribution from gluconeogenesis and tricarboxylic acid (TCA) cycle towards acetol formation to maintain NADPH/NADP^+^ balance during nitrogen limitation.

## Methods

### Strain, medium, and culture conditions

*E. coli* BW25113 [29] was used as a parent strain for engineering the acetol-producing whole-cell biocatalyst *E. coli* B4. The strain was modified via λ red recombineering and P1 transduction by deleting *ldhA* (lactate dehydrogenase)*, poxB* (pyruvate dehydrogenase), and the *pta-ackA* (phosphate acyltransferase, acetate kinase) operon to decrease byproduct formation. Further, *gloA* (lactoylglutathione lyase) and the *fnr* regulon (fumarate and nitrate reductase) were replaced with a chloramphenicol and a kanamycin resistance cassette, respectively, to enhance the availability of methylglyoxal (precursor of acetol). These modifications were partially described by Clomburg et al. [25]. Further, the engineered strain was laboratory-evolved for growth on glycerol, resulting in an increased glycerol uptake rate. Enabling production, *E*. *coli* B4 carries a plasmid (pTrcHis2B backbone, Trc/lac promotor, ampicillin resistance cassette, replicon: PBR322 ori) bearing two genes [methylglyoxal synthase (MGS, *mgsA*), aldehyde oxidoreductase (AOR, *yqhD*)], as partially described in a previous study [25].

Cryopreserved cells (− 80 °C, 20% v/v glycerol) were recovered on lysogeny broth (LB) agar plates containing the respective antibiotics for 24 h at 30 °C. A single colony was transferred into 10 mL LB medium supplemented with kanamycin (50 mg L^−1^), ampicillin (100 mg L^−1^), and chloramphenicol (12 mg L^−1^) for the first pre-culture. The culture was incubated at 30 °C and 200 rpm for 6–8 h to reach a sufficiently high optical density between 3 and 6. A volume of 500 μL was subsequently transferred to 50 mL modified M9 minimal medium, including respective antibiotics with 10 g L^−1^ glycerol as a carbon source for the second pre-culture. It was cultivated in 500 mL shake flasks at 30 °C and 200 rpm for 12–16 h. The composition of the modified M9 medium [30] supplemented with naturally labelled or 2-^13^C labelled glycerol had the following composition per liter: 160 mmol glycerol; 2 g Na_2_SO_4_·10H_2_O; 2.68 g (NH_4_)_2_SO_4_; 1 g NH_4_Cl; 1.46 g K_2_HPO_4_; 0.4 g NaH_2_PO_4_·2H_2_O; 0.25 g MgSO_4_·7H_2_O; 22 mg CaCl_2_·2H_2_O; 0.27 mg ZnSO_4_·7H_2_O; 0.15 mg MnSO_4_·H_2_O; 30.2 mg Na–EDTA; 0.24 mg CuSO_4_·5H_2_O; 24.1 mg FeCl_3_·6H_2_O and 0.27 mg CoCl_2_·6H_2_O. The final pH value of the medium was 7.1.

Cultivations in stirred-tank reactors (STRs) were performed using a BioFlo 3000 (New Brunswick Scientific Co., Inc.) with a working volume of 1.25 L modified M9 medium. The cultivation conditions were set to a temperature of 30 °C, a pH value of 6.8 ± 0.1 (adjusted with 5 M NaOH), agitation at 500 rpm, and a constant aeration rate of 1 vvm. The initial glycerol concentration was 15 g L^−1^, and the bioreactor was inoculated to an optical density of 0.1. The fermentation parameters (dissolved oxygen, pH, temperature, stirrer speed, off-gas) were monitored using BioCommand (New Brunswick Scientific Co., Inc.). The dissolved oxygen level was maintained at or above 40% via cascaded agitation. Growth was monitored regularly by measuring the optical density at a wavelength of 600 nm using a spectrophotometer (Ultrospec 10 Cell density meter, Amersham Life Sciences). All collected cultivation data resulted from fermentations in STRs.

### Analysis of energy and redox cofactors

During the cultivation, 4 mL of cell broth was sampled directly into 1 mL of perchloric acid and thoroughly mixed in an overhead shaker for 15 min at 4 °C. At acidic pH values, the oxidized cofactors (*i.e.*, NAD^+^ and NADP^+^) are stable, whereas their reduced forms are unstable [31]. Thereby, any inaccuracy of cofactor quantification due to oxidation is avoided. Subsequently, the sample was neutralized using appropriate amounts of 1 M K_2_HPO_4_ and 5 M KOH while shaking in ice water. The neutralized sample was centrifuged at 4,696 g at 4 °C, and the supernatant was stored at –20 °C. HPLC-UV analysis was performed to quantify energy- and redox cofactors (Beckman System Gold, column: LiChrospher RP-18; length: 25 cm; diameter: 4*.*6 mm), applying an existing protocol [32]. A gradient profile of two buffers was designed. As buffer A 0.1 M KH_2_PO_4_/K_2_HPO_4_ at a pH value of 6 supplied with 4 mM tetrabutylammonium hydrogen sulfate (TBAHS) and 0.5% (v/v) methanol was used. Buffer B consisted of 70% (v/v) buffer A plus 30% (v/v) methanol and was adjusted to a pH value of 7.2 using 5 M KOH. For the first 3.5 min, 100% buffer A was applied, gradually decreasing to 0% at 43.5 min. After that, buffer B was kept at 100% for 7.5 min, after which a gradual switch back to buffer A (100% at 56 min) was applied. The flow rate and the temperature were kept constant at 1 mL min^−1^ and 25 °C, respectively. The injected sample volume was 20 µL.

### Analysis of extracellular metabolites

Approximately 200 μL of cell-free filtrate was obtained using an in-line filtration probe (FISP D-Series, Flownamics, 0.2 µm pore size) connected to the sampling port of the bioreactor. HPLC-UV-RI analysis was performed on a Beckman System Gold using a 166 UV detector and a refractive index detector. The metabolites were separated with an isocratic method using 5 mM H_2_SO_4_ with a flow rate of 0.6 mL min^−1^ at a temperature of 75 °C (organic acid resin column, polystyrol-divinylbenzol copolymer (dimensions 300 × 8.0 mm), CS Chromatographie Service, GmbH).

Ammonium concentrations were estimated using the test kit QUANTOFIX (Macherey–Nagel GmbH & Co. KG). For quantitative measurement of ammonium concentrations, the Willis method was adopted [33]. 50 μL of cell-free medium was added to a 24-well plate along with 1 mL of reagent solution, followed by quick mixing with 0.25 mL hypochlorite. After 15 min, the absorbance was measured at 685 nm. The calibration standards (5–50 mg L^−1^ of NH_4_^+^) were also processed as described above.

### Analysis of intracellular metabolites

Rapid sampling was performed using a custom-made semi-automated device with minimal dead volume. 1.5 mL of broth was withdrawn from the fermenter in less than 1 s and injected directly into 9 mL of pure methanol at –80 °C, followed by instant vortexing for immediate quenching of metabolic activity [34]. The quenched samples were centrifuged at 4696*g* for 5 min at -10 °C. After decanting the supernatant, the intracellular metabolites were extracted from the cell pellet.

For metabolite extraction, the biomass pellet was resuspended in 500 μL of 80% (v/v) methanol in tightly screwed shut and sealed tubes and placed in a heating block set at 95 °C for 5 min. Immediately thereafter, the reaction tubes with extracted samples were cooled to –20 °C. Afterwards, the samples were centrifuged at 4696*g* for 10 min at –10 °C and stored at -80 °C until analysis [34].

Intracellular metabolites present in the metabolite extract were analyzed by following a two-step derivatization procedure [34] and GC-MS analysis protocol according to the method described by Dunn et al. [35]. To an extract volume of 200 µL, 50 µL of *O*-methylhydroxylamine hydrochloride (20 mg mL^−1^ dissolved in pyridine) and 4 µL of the internal standard erythritol (final concentration of 40 μM) was added and thoroughly mixed. The mixture was dried overnight using a vacuum concentrator (ScanSpeed MaxiVac, LaboGene A/S) at 30 °C. In the first derivatization step, 50 μL of *O*-methylhydroxylamine hydrochloride (20 mg mL^−1^ dissolved in pyridine) was added to the dried sample, thoroughly mixed, and incubated at 80 °C for 15 min at vigorous shaking. In the second step, 50 μL of MSTFA (*N*-methyl-*N*-trimethylsilyltrifluoroacetamide, CS-Chromatographie Service GmbH) was added manually to all samples at the same time, thoroughly mixed and incubated at 80 °C for another 15 min. The derivatized samples were analyzed on a gas chromatography-mass spectrometry (GC-MS/MS) system consisting of a TRACE™ GC Ultra (Thermo Scientific™ TRACE™ 1300 GC) coupled to a triple quadrupole mass spectrometer (Thermo Scientific™ TSQ 8000™, Thermo Fisher Scientific GmbH) with electron impact ionization (Thermo Fisher Scientific GmbH). Chromatographic separations were performed using a fused silica capillary column with a 1,4-bis (dimethylsiloxy) phenylene dimethyl polysiloxane matrix (Thermo Scientific™ TraceGOLD TG-5SilMS; length: 30 m; inner diameter: 0.25 mm; film thickness, 0.25 µm). The temperature program for separation and the operating conditions of the mass spectrometer were followed according to a protocol described earlier [35]. The raw GC-MS/MS data were transformed into mass distribution vectors and were corrected for unlabeled biomass from the inoculum and the natural abundance of heavy isotopes using iMS2Flux [36].

### Biomass hydrolyzation and amino acid derivatization

Biomass samples (equivalent to 0.3 mg cell dry weight) were centrifuged and washed twice with 0.9% (v/v) NaCl. The pellet was resuspended in 150 μL 6 M HCl and incubated for 6 h at 105 °C in a sealed tube placed in a fume hood. Subsequently, the liquid was evaporated overnight at 85 °C in a fume hood. The dried hydrolysate was resuspended in 30 μL acetonitrile and 30 μL MBDSTFA (*N*-methyl-*N*-tert-butyldimethylsilyl-trifluoroacetamide) in a conical glass vial, mixed for 15 s, and incubated at 85 °C for 1 h. The samples were analyzed immediately on the gas chromatography-mass spectrometry (GC-MS) system consisting of a TRACE™ GC Ultra and a quadrupole MS with electron impact ionization (Thermo Fisher Scientific) equipped with a TraceGOLD TG-5SilMS fused silica column (length, 15 m; inner diameter, 0.25 mm; film thickness, 0.25 μm). The GC-MS was operated as described by Schmitz et al. [37] with a constant gas flow rate of 1 mL min^−1^ of helium and a split ratio of 1/15. The injector temperature was set to 270 °C, and the column oven program comprised an initial temperature of 140 °C for 1 min and a temperature ramp with a rate of 10 °C min^−1^ to a final temperature of 310 °C with a hold time of 1 min. The raw GC-MS data were transformed into mass distribution vectors and were corrected for unlabeled biomass from the inoculum and the natural abundance of heavy isotopes using iMS2Flux [36].

### Targeted metabolic flux ratio analysis

Metabolic flux ratios were estimated according to the methods described by Kogadeeva et al. [38], using the software SUMOFLUX, implementing the ^13^C-labelling patterns simulated by the software INCA version 1.6 [39]. The metabolic network model (Fig. 1) used along with carbon atom transitions is described in Supplementary Files S1 and S2. The model was adapted [40] and modified according to the genetic modifications of the engineered acetol-producing *E. coli* B4. Flux constraints (normalized to the glycerol uptake) were specified according to the metabolites detected and quantified in the cell-free filtrate during growth and the nitrogen-limited phase.

### Metabolic flux analysis

A metabolic network (Supplementary File S2) based on a SUMOFLUX model was generated using COBRApy (0.11.3) [41]. Exchange bounds were set to mean values for the time points in each phase, and upper/lower bounds to equivalent ranges based on SUMOFLUX constraints. Ratio constraints were represented as

$$N_{i} - R_{i} D_{i} + A_{i} = 0$$

in the model, where ${N}_{i}$ and ${D}_{i}$ represent the sum of fluxes for reactions used in the nominator and denominator, respectively, ${R}_{i}$ is the mean ratio estimated with SUMOFLUX, and ${A}_{i}$ is an auxiliary variable to be used for the optimization problem. A flux solution was obtained through minimization for

$$\mathop \sum \limits_{i} \left( {1 - E_{i} } \right)\left( {A_{i} } \right)^{2}$$

where ${E}_{i}$ is the phase’s mean absolute error (MAE) for the ratio. The flux distribution was visualized on a metabolic map using Escher (1.7.0-beta.4) [42].

## Results

### Nitrogen limitation triggers acetol biosynthesis from glycerol in engineered *E.* *coli*

The acetol-producing strain *E. coli* B4 is based on a chassis subjected to adaptive laboratory evolution (ALE) to enhance glycerol uptake and conversion. Whole-genome re-sequencing revealed an acquired single point mutation in the gene encoding the glycerol kinase (GlpK), resulting in an amino acid substitution from aspartate to valine at position 73 (*glpK-D73V*). Although the distinct effect of the mutation has not been investigated in this study, several previously detected mutations in *glpK*, including the one identified here, have been shown to increase affinity for glycerol [43], and thereby, a similar effect is suggested in the presented case. Another single point mutation was detected in the gene encoding the oxidative and nitrosative stress transcriptional regulator OxyR. In its active form, caused by oxidation by hydrogen peroxide, it activates the transcription of genes, enabling *E. coli* to defend against reactive oxygen species (ROS) [44, 45]. The point mutation identified here causes an amino acid exchange from leucine to glutamine at position 224 (*oxyR-L224Q*). In its reduced form, Leu-224 hydrophobically interacts with Leu-200 to stabilize the protein [46]. Among other structural modifications, this hydrophobic interaction needs to be overcome for transition to the active form. Therefore, an exchange to the polar amino acid glutamine might indicate a facilitated activation and a potential advantage of the strain in response to ROS. However, this was not examined in this study. Few further point mutations were detected, mainly located in intergenic regions and likely representing artifacts caused by metabolic engineering (Supplementary File S7).

**Fig. 2:**
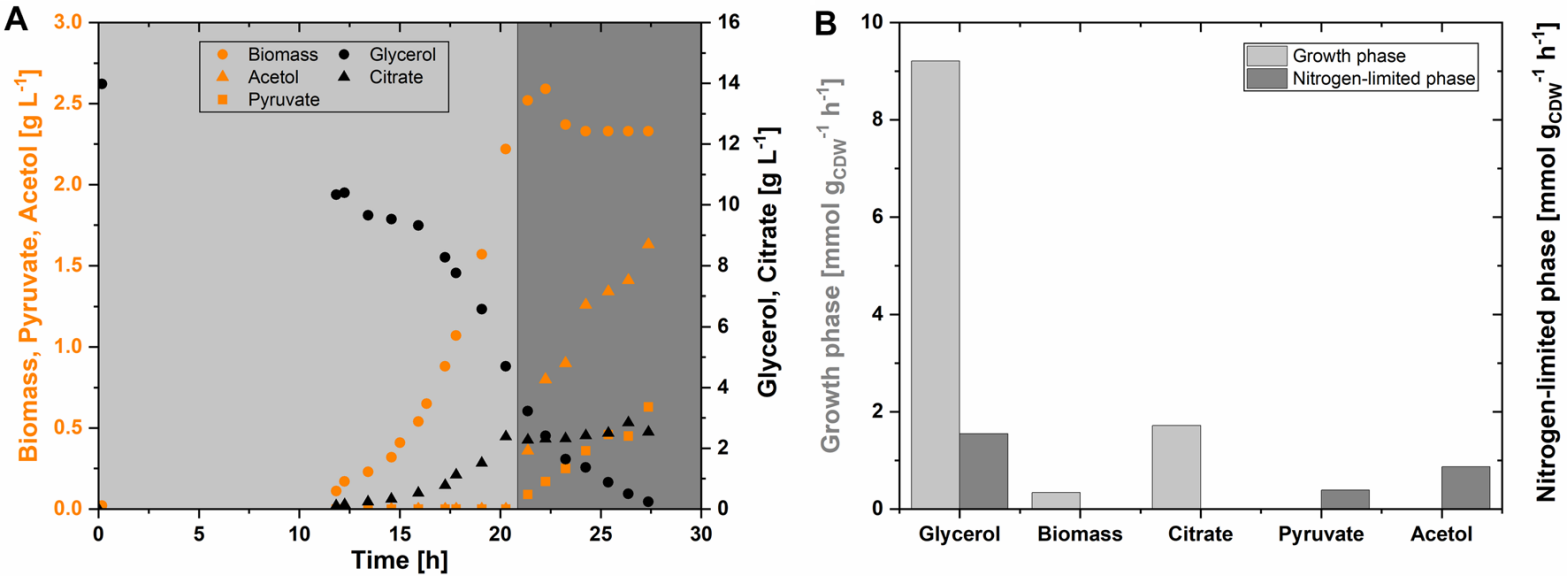
Growth profile (**A**) and physiology (**B**) of *E. coli* B4 on 100% labeled 2-^13^C glycerol. In both figures, the areas shaded light gray and dark gray represent the growth phase and nitrogen limitation phase, respectively. For the analysis of mass isotopomer distributions, samples were taken from the growth phase at 13.75 h, 15.00 h, 16.33 h, and 17.80 h and from the nitrogen-limited phase at 24.25 h, 25.37 h, 26.37 h, and 27.37 h (**A**). Orange circles, triangles, and squares represent the measured biomass, acetol, and pyruvate concentrations, respectively. The black circles and triangles represent the measured glycerol and citrate concentrations, respectively

The cultivation medium was designed with a C/N ratio of 7 to trigger nitrogen limitation when about 65% of the carbon is consumed, resulting in no biomass formation after an exponential growth on glycerol. Whereas no acetol was produced in the exponential growth phase, the growth arrest forced by nitrogen depletion initiated the production of acetol (Fig. 2A). During the exponential growth phase, the cells reached a maximum biomass concentration of 2.5 g_CDW_ L^−1^ with a growth rate of 0.34 h^−1^. While biomass, citrate, and carbon dioxide are the major products in the growth phase, pyruvate and acetol are mainly produced during nitrogen limitation. The strain *E*. *coli* B4 reached a maximum acetol titer of 1.63 g L^−1^ with a yield of 0.53 mol_Acetol_ mol_Glycerol_^−1^ (0.43 g_Acetol_ g_Glycerol_^−1^) and a productivity of 0.25 g_Acetol_ L^−1^ h^−1^ (in about 6.5 h from the onset of nitrogen limitation) or 0.06 g_Acetol_ L^−1^ h^−1^ (in about 27 h including the phases of biomass formation and nitrogen limitation). The metabolic activity of cells defined by the specific glycerol uptake rate declined under nitrogen limitation to about 18% compared to the exponential growth phase (Fig. 2B). In this engineered acetol-producing strain, pyruvic acid is formed as the major by-product during the resting but active phase of glycerol conversion with a yield of 0.25 mol_Pyruvate_ mol_Glycerol_^−1^. These results elucidate the physiological significance of nitrogen limitation towards the biosynthesis of acetol. When using glycerol as the sole carbon source, the flux distribution, especially during nitrogen limitation, is of interest to optimize acetol production further.

### Metabolic fluxes of engineered *E.* *coli* during growth on 2-^13^C glycerol

**Fig. 3:**
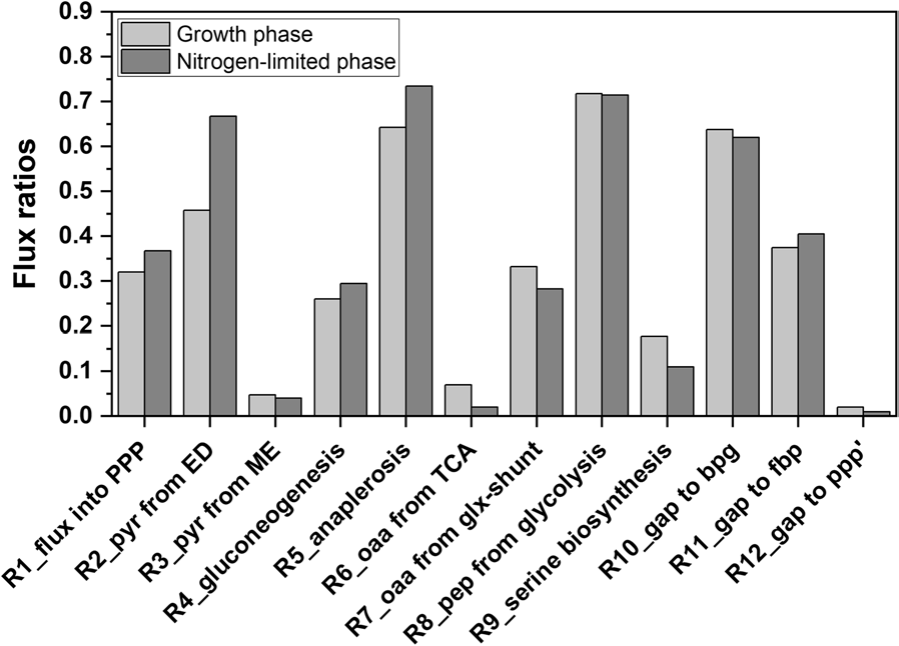
The SUMOFLUX estimates of metabolic flux ratios. The fluxes represent the origin of metabolic intermediates during the growth and nitrogen-limited phases of *E. coli* B4 on 2-^13^C glycerol. The light gray bars represent the growth phase, and the dark gray bars represent the nitrogen-limited phase. The definition of flux ratios is described in Supplementary File S1, and the mean absolute error (MAE) is given in Supplementary File S4

**Fig. 4:**
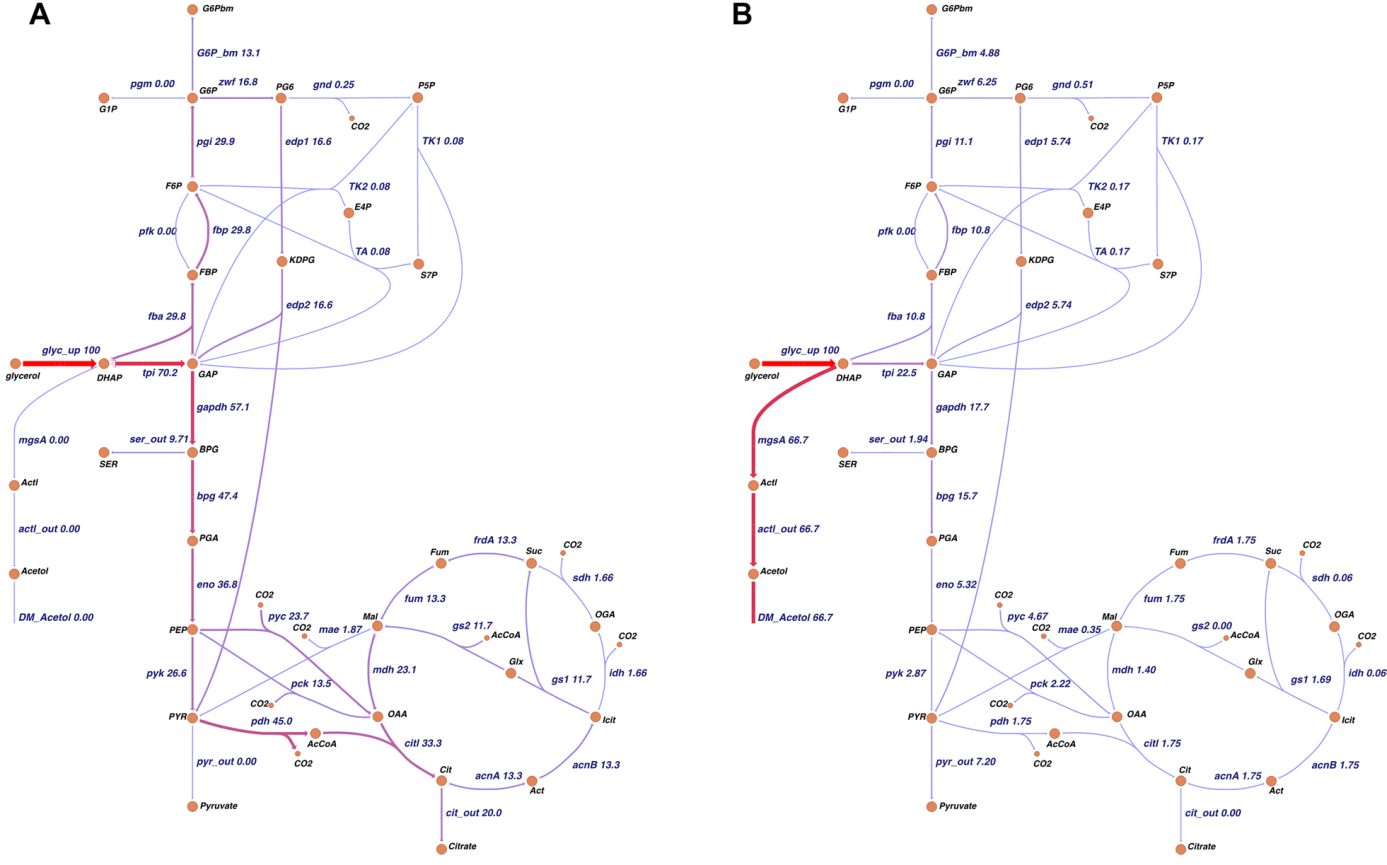
Central carbon metabolism and heterologous pathway for the production of acetol as represented in the metabolic network model. Metabolic flux distribution of acetol-producing engineered *E. coli* B4 during growth (**A**) and the nitrogen-limited phase (**B**) on 100% 2-^13^C glycerol as carbon and energy source. Metabolites: DHAP, dihydroxyacetone phosphate; GAP, glyceraldehyde 3-phosphate; FBP, fructose 1,6-bisphosphate; F6P fructose 6-phosphate; G1P, glucose-1-phosphate; G6P, glucose-6-phosphate; PG6, 6-phosphogluconate; R5P, ribose 5-phosphate; S7P, sedoheptulose 7-phosphate; E4P, erythrose 4-phosphate; KDPG, 2-keto-3-deoxy-6-phosphogluconate; BPG, 3-phosphoglyceric acid; SER, serine; PGA, 2-phosphoglyceric acid; PEP, phosphoenolpyruvate; PYR, pyruvate; Mal, malate; Fum, fumarate; Suc, succinate; OGA, 2-oxoglutaric acid; Icit, isocitrate; Act, aconitate; Cit, citrate; OAA, oxaloacetate; Glx, glyoxylate; AcCoA, acetyl-coA; CO_2_, carbondioxide. The numbers given represent fluxes normalized to the glycerol uptake, and the thickness and coloring of the arrows indicate the strength of the flux. Absolute and normalized fluxes are presented in Supplementary File S5. The expected labeling pattern is represented in [47]

To understand the significant change in substrate, biomass, and product or by-product profile in the presence or absence of nitrogen, ^13^C metabolic flux analysis was performed with 2-^13^C glycerol as the only carbon and energy source. During growth on 2-^13^C glycerol, samples were taken at periodic intervals to monitor growth and metabolite profiles (Fig. 2A). The mass isotopomer distributions (MID) of free intracellular metabolites and proteinogenic amino acids were measured and used for metabolic flux ratio analysis with the software SUMOFLUX (refer to Materials & Methods). A pseudo-labeling steady-state was assumed when calculating the average fractional labeling of each metabolite from the respective metabolite MID (Supplementary File S3), as the fractional labeling patterns did not differ significantly in various samples taken at different stages within the exponential growth phase or the nitrogen-limited phase, respectively. With the labeling patterns of metabolites, a total of twelve metabolic flux ratios were estimated using SUMOFLUX (Fig. 3, Supplementary File S4). Subsequently, absolute intracellular fluxes were estimated from the obtained metabolic flux ratios on 2-^13^C glycerol during the growth and nitrogen-limited phase (Fig. 4, Supplementary File S5).

During the growth phase, most of the carbon flux is estimated to be directed toward gluconeogenesis (approximately 60%), as indicated by the conversion of DHAP and GAP, which are directly derived from glycerol to FBP (Fig. 4A). The produced glucose-6-phosphate was used partly for biomass synthesis (44%). The rest entered the oxidative pentose phosphate pathway (56%) for NADPH synthesis. About 98% of the flux from 6-phosphogluconate was channeled through the Entner–Doudoroff (ED) pathway, leaving a major pool of pyruvate and GAP molecules through this origin, which was also observable from the ‘Pyruvate to ED’ flux ratio of 0.46 and a low ‘GAP from PPP’ flux ratio of 0.02 (Fig. 3). Thus, 57% of the carbon was channeled towards the lower part of glycolysis. Apart from the carbon flux towards serine biosynthesis and the fraction of PGA used for biomass synthesis, 64% of the carbon flux entered the phosphoenolpyruvic acid (PEP)-TCA/glyoxylate cycle for ATP, NADH, NADPH, and biomass precursor generation. The combined fraction of pyruvate originating from the ED pathway, the pyruvate kinase, and the malic enzyme was used for NADH, acetyl-CoA (AcCoA), and CO_2_ generation via the pyruvate dehydrogenase. The citrate converted from the pools of oxaloacetate (OAA) and AcCoA via the citrate synthase was also found to be secreted during the exponential growth phase on glycerol. Only 40% of the flux from citrate was channeled into the TCA cycle. The major fraction of the carbon flux from isocitrate (88%) was found to be re-routed to the glyoxylate shunt, and OAA was further substantially replenished by anaplerosis (0.64, OAA from PEP, R5_anaplerosis, Fig. 3). The minor flux through the isocitrate and 2-oxoglutarate dehydrogenase indicates that a significant amount of carbon is used for NADH and ATP generation rather than NADPH generation in the PEP-glyoxylate cycle. Finally, the PEP-glyoxylate cycle is completed by the PEP carboxykinase, with about 29% of the total OAA converted to PEP by this enzyme.

### Decreased flux through the central carbon metabolism under nitrogen limitation favors cofactor availability for acetol formation

Under nitrogen limitation, the glycerol uptake rate was reduced to 1.54 mmol g_CDW_^−1^ h^−1^, representing a decrease of about 82% compared to the growth phase. Significant changes were observed in the metabolic fluxes during the nitrogen-limited phase compared to the abovementioned growth phase. Notably, under nitrogen limitation, 67% of the glycerol flux from DHAP was redirected to acetol biosynthesis (Fig. 4B). The carbon flux towards gluconeogenesis was estimated at 22%, accounting for 49% of the available carbon, which is not used for acetol biosynthesis. Such strong re-routing to product formation substantially reduced carbon flux to the remaining parts of central carbon metabolism. As a result, the fraction of pyruvate originating from lower glycolysis or pyruvate kinase is lower when nitrogen is limiting compared to the growth phase. This is reflected in an about 45% increase in the fraction of pyruvate originating from the Entner–Doudoroff (ED) pathway (Fig. 3).

Further, the flux from pyruvate into the TCA cycle via pyruvate dehydrogenase is reduced under nitrogen limitation, observed from the extracellular accumulation of pyruvate and in the decrease of oxaloacetate (OAA) fraction from TCA cycle (71% decrease) and from the glyoxylate shunt (15% decrease) compared to the growth phase. The demand for OAA is compensated by a 15% increase in the anaplerotic reaction. Although there is a slight increase of 15% in the flux towards PPP, a 50% decrease was observed in the origin of GAP from the PPP under nitrogen limitation.

**Fig. 5:**
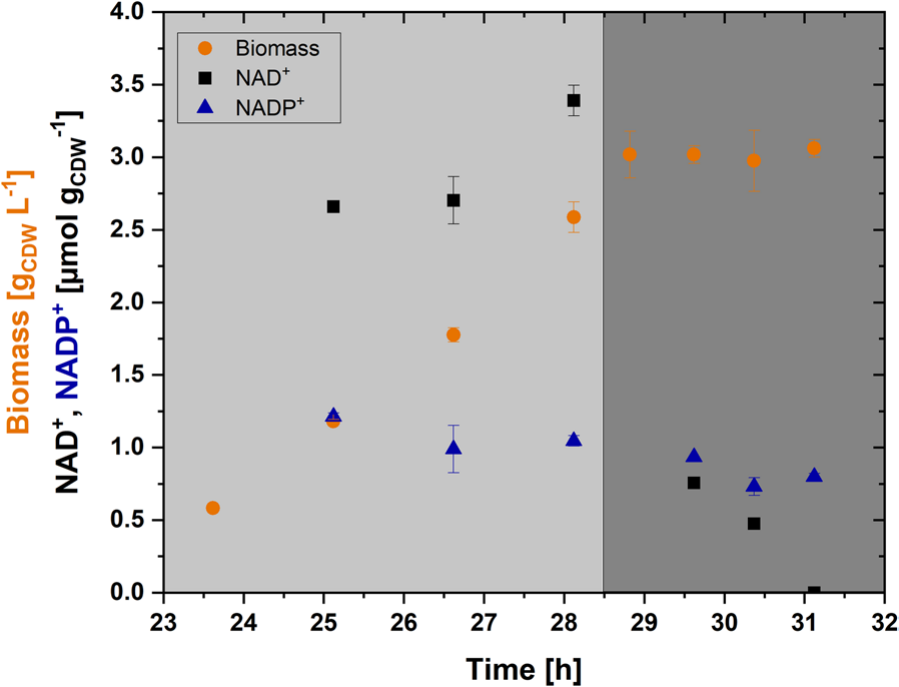
Change in concentration of NAD^+^ and NADP^+^ during the transition from growth to nitrogen-limited phase in *E. coli* B4. The shaded light gray and dark gray areas represent the growth and nitrogen limitation phases, respectively. Orange circles, black squares, and blue triangles represent biomass, NAD^+^, and NADP^+^, respectively. The error bars denote the standard deviation in the measurements obtained from technical triplicate samples

In general, the flux through the PEP-glyoxylate cycle was highly reduced under nitrogen limitation compared to the growth phase, signifying a reduction in cofactor generation through this route. This also indicates that the demand for NADPH for acetol biosynthesis and maintenance reactions was at least partially compensated via the PPP under nitrogen limitation. Further, to understand the changes in cofactor requirement under nitrogen limitation, the concentrations of cofactors (NAD^+^ and NADP^+^) were measured during the growth and nitrogen-limited phase (Fig. 5). Before the transition to nitrogen limitation, the concentration of NAD^+^ was 3.39 ± 0.11 µmol g_CDW_^−1^, while shortly after the transition to nitrogen limitation, the NAD^+^ concentration decreased to 0.76 ± 0.02 µmol g_CDW_^−1^. Within 1.5 h of further cultivation, no NAD^+^ was detected, indicating an excessive requirement of NAD^+^ in parts of the metabolism, such as in the conversion of glycerol-3-phosphate to DHAP, lower parts of the glycolysis, and the TCA cycle under nitrogen limitation. In contrast, the normalized concentration of NADP^+^ decreased to a lesser extent in the nitrogen limitation phase compared to the growth phase, indicating that the requirement of NADPH in the conversion of methylglyoxal to acetol is in equilibrium with the NADPH regeneration reaction of the oxidative PPP. Consistent with the measured concentrations of cofactors, the estimated net rates of NADH and NADPH (Supplementary File S6) show that a balance in NADPH/NADP^+^ regeneration but not in NADH/NAD^+^ regeneration exists. As these two independent methods (measurement of cofactor concentrations and production/consumption rates based on flux analysis) agree, a maintenance of the NADPH/NADP^+^ balance under nitrogen limitation can be concluded. As the anabolic demands for biomass synthesis, thus NADPH sinks, are low compared to the growth phase, acetol biosynthesis is favored under nitrogen limitation to uphold the NADPH/NADP^+^ balance.

## Discussion

In this study, we show that acetol production from glycerol is triggered by the onset of nitrogen limitation in the engineered *E. coli* B4 strain. As demonstrated, this is due to a metabolic effect. The biosynthetic pathway for acetol requires NADPH, and its replenishment potentially represents a bottleneck in efficient acetol production. In a previous study, Yao et al. enhanced NADPH availability by overexpressing both the ATP-dependent NAD kinase (NadK) converting NAD^+^ to NADP^+^ and the membrane-bound transhydrogenase (PntAB) transferring a hydride from NADH to NADP^+^ [27]. This approach allowed the production of 2.81 g L^−1^ acetol from 10 g L^−1^ glycerol in shake flask cultivations during the growth phase. In the presented study, acetol was produced only after the onset of nitrogen depletion and cessation of biomass formation, thus decoupling growth and production. Under this condition, the carbon flux was found to be significantly redirected from the central carbon metabolism to the acetol production pathway. Quantification of redox equivalents revealed a shortage of NADH under N-limited conditions, however, the cells were able to balance NADPH concentrations.

NADPH homeostasis has been shown previously to be crucial for cells at limiting nutrient conditions [48]. The absence of biomass formation further limits the usage of NADPH in the anabolic reactions, resulting in an excess of NADPH. Thus, an alternative sink is needed to achieve homeostasis. In the presented study, this sink is represented by the NADPH-dependent aldehyde oxidoreductase, converting methylglyoxal to acetol. In this argumentation, the lack of acetol production during growth may be explained by the predominant use of NADPH for biomass synthesis. While the final titer of acetol (1.63 g L^−1^) was lower compared to the reported values (2.81 g L^−1^ in 72 h) [27], the production occurred from the onset of nitrogen starvation until glycerol depletion within 6.5 h, leading to improved productivity (0.25 g_Acetol_ L^−1^ h^−1^, only production phase or 0.06 g_Acetol_ L^−1^ h^−1^ entire cultivation; this study) compared to 0.04 g_Acetol_ L^−1^ h^−1^ [27]). Limiting biomass concentrations increases the yield of biochemically derived products closer to the theoretical maximum and significantly reduces manufacturing costs [49]. However, this approach requires appropriate selection strategies to ensure sustained metabolic activity in the absence of biomass formation and limiting nutrients [50]. Considering the nitrogen-limited phase, specific production rates of 0.11 g_Acetol_ g_CDW_^−1^ h^−1^ were reached, demonstrating the potential of the constructed whole-cell biocatalyst. Potentially, feeding strategies or reusing produced biomass for resting cell production could further enhance yield and productivity.

In *E. coli*, biochemical production was found to be enhanced under growth limitation by inhibiting biomass formation either by limiting nutrients or by adding growth inhibitors [51, 52]. In the case of mevalonate-producing *E. coli*, the amount of available NADPH for product formation was identified to be higher under sulfur limitation than nitrogen or magnesium limitation, which was also reasoned by a reduced flux through the TCA cycle [52]. During fermentative glycerol utilization, *E. coli* was found to possess multiple measures for achieving redox balance depending on environmental conditions such as pH, with proton reduction to hydrogen (H_2_) via a reversible hydrogenase critical under alkaline conditions [53]. A recent study demonstrated a simple strategy to enable fermentative growth of *E. coli* on glycerol in a defined minimal medium with acetate as a co-substrate to resolve a redox imbalance [54]. Creating additional energetic demand by overexpressing an ATP-hydrolyzing ATPase seems to be another promising approach [55, 56].

In *E. coli* B4, although major by-products like acetate were avoided by deleting the acetate production pathways, citrate was found to be produced as a by-product during the growth phase. In contrast, pyruvate was produced during the nitrogen-limiting phase. While the accumulation of pyruvate in the nitrogen-limitation phase can be explained by the decreased pyruvate dehydrogenase flux observed in ^13^C-MFA, citrate formation cannot be reasoned by the flux results of this study. However, *E. coli* has been observed to secrete significantly more citrate when grown on glycerol or acetate compared to glucose as the sole carbon source [57]. Further research is needed to develop metabolic engineering strategies to avoid carbon loss due to citrate production. Pyruvate synthesis might be reduced by down-regulating the expression of the gene encoding for the glyceraldehyde 3-phosphate dehydrogenase (*gap*), potentially enabling enhanced product formation, as described previously [58]. Further metabolic engineering of the strain can be directed to synthesizing 1,2-propanediol, a commercially valuable product demonstrated in previous studies [17, 25, 59–61]. Conversion of acetol to 1,2-propanediol in *E. coli* B4 can be driven by the enzyme glycerol dehydrogenase (GldA), which requires NADH as a cofactor. The glycerol dehydrogenase is native to *E. coli*, theoretically enabling it to produce 1,2-propanediol. However, the NADH supply might not be sufficient under nitrogen-limited conditions, allowing acetol production. As GldA additionally catalyzes the oxidation of glycerol to dihydroxyacetone (DHA), an overexpression of *gldA*, encoding a NAD^+^-dependent glycerol dehydrogenase, can potentially resolve the NADH shortage, thus enabling the strain to achieve both NADH and NADPH homeostasis and ultimately resulting in the production of 1,2-propanediol. Further, to produce and tolerate high concentrations of 1,2-propanediol, adaptive laboratory evolution might be necessary. A few key mutations in *fuc* regulon and 1,2-propanediol catabolism were reported in previous laboratory evolution studies with *E. coli* [62, 63].

## Conclusion

The results of this study showed that engineered *E. coli* can produce acetol under nitrogen limitation. The absence of biomass formation under these limiting conditions was favorable for the cells to balance NADPH concentrations via acetol biosynthesis by the NADPH-dependent aldehyde oxidoreductase. While pyruvate was found to be formed as a major byproduct under nitrogen-limited growth conditions in this study, it is essential to recognize that pyruvate is one of the key metabolic intermediates for the production of a range of bio-based chemicals by rational metabolic engineering strategies approaches [64, 65]. These findings highlight the importance of further engineering the strain towards sustainable production processes based on glycerol as a feedstock.

## Supplementary Information

Supplementary Material 1.

Supplementary Material 2.

Supplementary Material 3.

Supplementary Material 4.

Supplementary Material 5.

Supplementary Material 6.

Supplementary Material 7.

## Data Availability

No datasets were generated or analysed during the current study.
